# Supplementary material for: Programming crack patterns with light in colloidal plasmonic films
Source: Nat Commun. 2024 Feb 7;15:1156. doi: 10.1038/s41467-024-45365-1 (PMC10850101; doi:10.1038/s41467-024-45365-1)
Supplement: Supplementary file 1 — Supplementary Information [file 41467_2024_45365_MOESM1_ESM.docx]

**Supplementary information**

**Programming cracks patterns with light in colloidal plasmonic films**

Fanny Thorimbert,^1^ Mateusz Odziomek,^2^ Denis Chateau,^3^ Stéphane Parola,^3^ Marco Faustini^1,4*^

**Affiliations**

1) Sorbonne Université, CNRS, UMR 7574, Chimie de la Matière Condensée de Paris, F-75005 Paris, France.

2) Colloid Chemistry Department, Max Planck Institute of Colloids and Interfaces, Am Mühlenberg 1, 14476 Potsdam, Germany

3) Ecole Normale Supérieure de Lyon, CNRS UMR 5182, Université Claude Bernard Lyon 1, Laboratoire de Chimie, 46 allée d'Italie, F69364 Lyon, France

4) Institut Universitaire de France

*Correspondence to marco.faustini@sorbonne-universite.fr


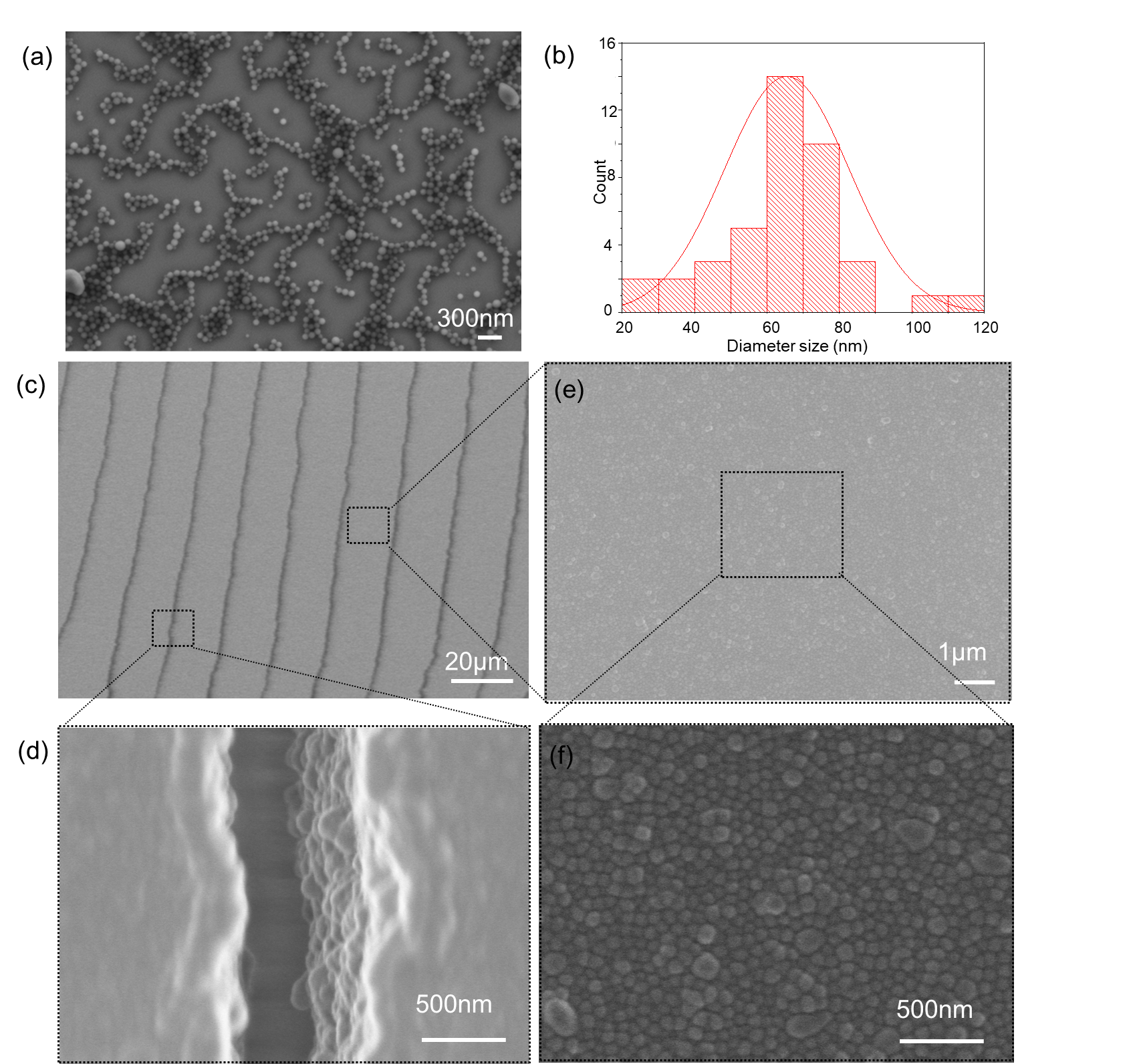


Supplementary Figure 1: SEM characterization of polystyrene cracked film. (a) SEM image of polystyrene nanoparticles and (b) size distribution. (c) SEM images of a cracked pattern with zoom in different zones of the sample (d), (e), (f).


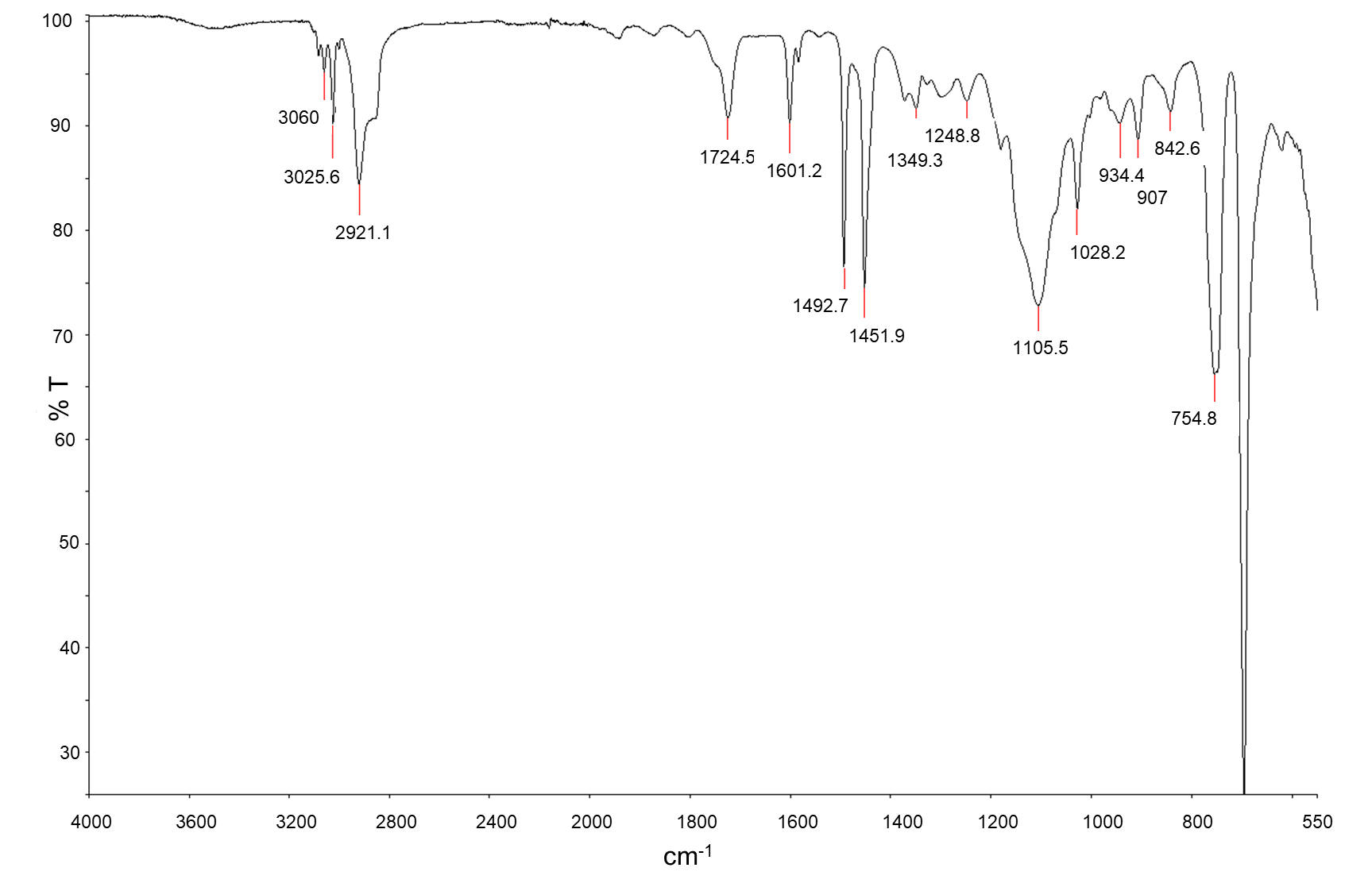


Supplementary Figure 2: Infrared spectroscopy of a dried polystyrene droplet. Absorption peaks at wave numbers 3060 and 3025.6 are due to aromatic C-H stretching vibration, whereas the absorption peaks at wave numbers 1601.2, 1492.7 and 1451.9 are due to aromatic C=C stretching vibration. These peaks prove the presence of benzene rings. In addition, the peaks at 754.8 and 694 correspond to C-H out-of-plane bending vibrations and indicate that there is only one substituent in the benzene ring. These are typical signatures coming from polystyrene. The peaks between 3000 and 2700 cm^-1^ are typically assigned to stretching vibrations of sp^3^ C-H which can be found in polystyrene and pluronic F127. The presence of the latter is additionally confirmed by relatively strong peaks between 1200-1000 cm^-1^, which are assigned to C-O vibrations. Source data are provided as a Source Data file.


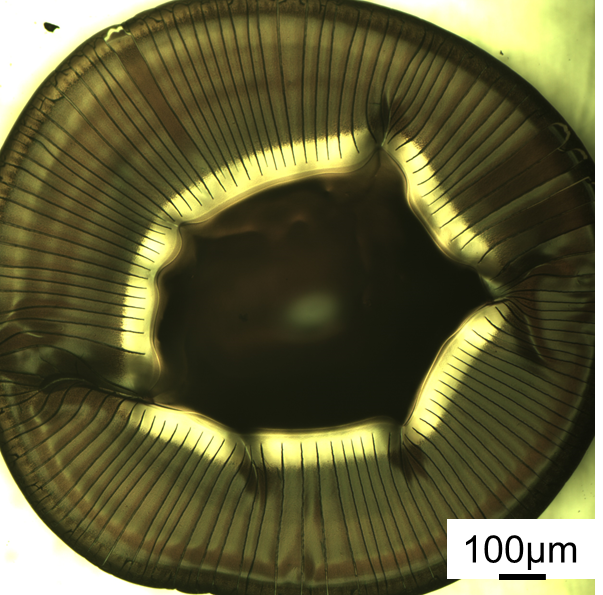


Supplementary Figure 3: Drying of a colloidal droplet. Optical microscopy image of a drying colloidal droplet with radial, evenly spaced cracks.


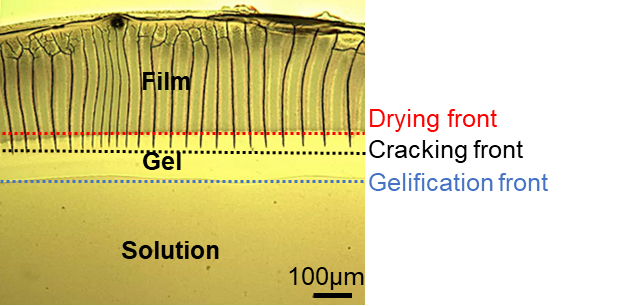


Supplementary Figure 4: Characterization of a drying colloidal film. This optical microscopy image shows the presence of three distinct zones, the colloidal solution, the colloidal gel filled with water and the dried film. The three zones are separated by the gelification front and the drying front. Cracks appear in the gel state, at a distance that is indicated as cracking front.


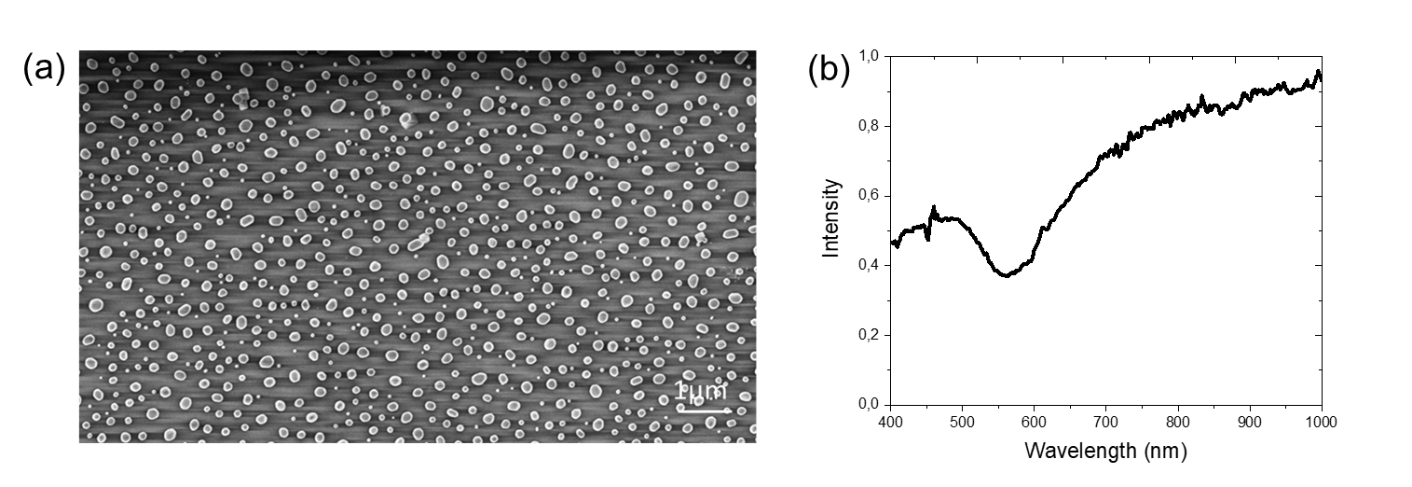


Supplementary Figure 5: Characterization of plasmonic, dewetted gold particles. (a) SEM image of dewetted gold particles on a silicon substrate, obtained by thermal treatment (450°C – 20mins) of a 10 nm metallized gold layer and (b) its transmittance spectrum. Source data are provided as a Source Data file for Figure (b).


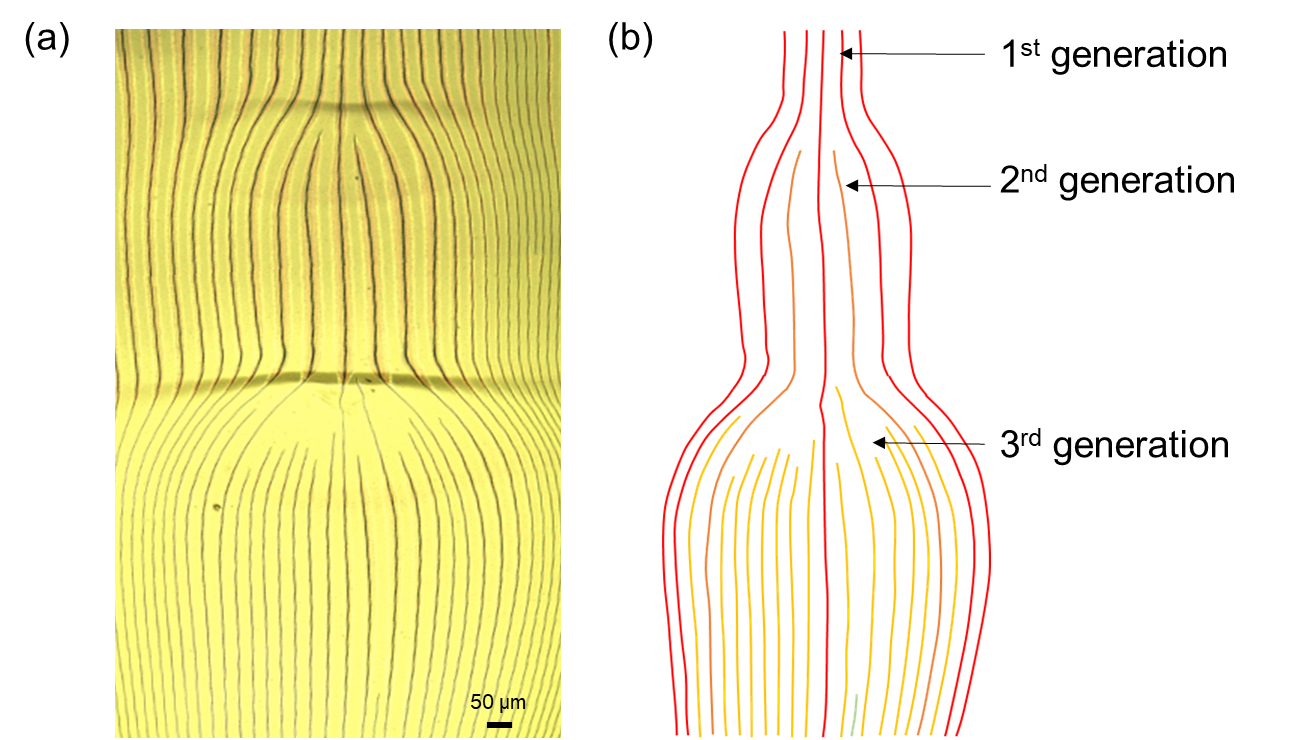


Supplementary Figure 6 : New generation of cracks. (a) Optical micrograph of crack deviation with the formation of new generations of cracks, (b) with the corresponding scheme.


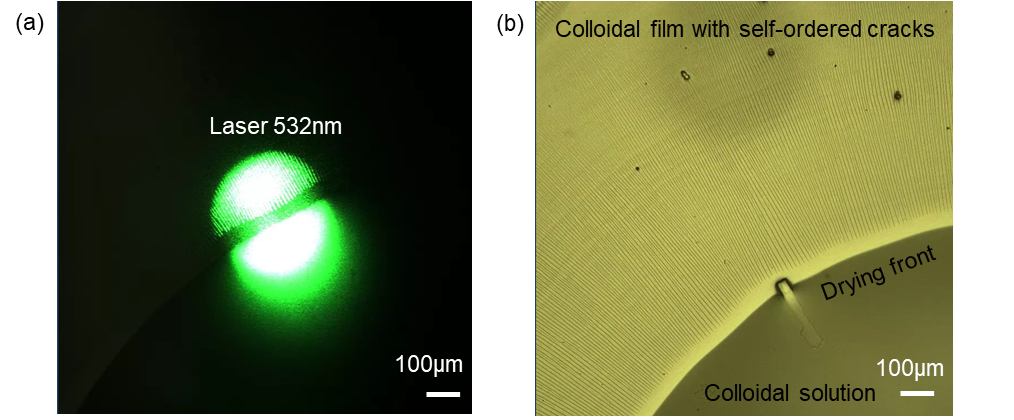


Supplementary Figure 7: Characterization of the crack-deviation on a non plasmonic substrate. Optical micrographs of a (a) drying colloidal droplet on a glass substrate without plasmonic particles illuminated by a 532nm laser. (b) No deviation of cracks occurs.


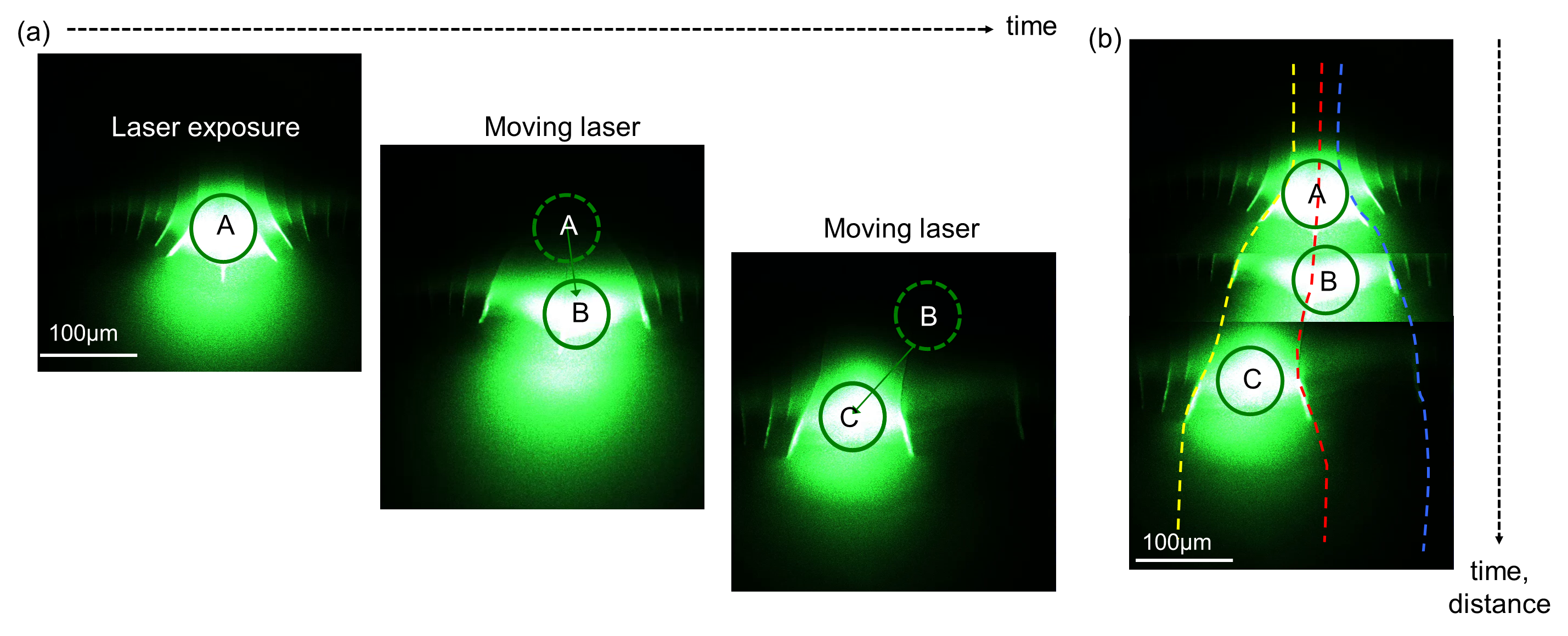


Supplementary Figure 8: Cracks escaping light. (a) Optical microscopy image of a drying colloidal droplet exposed to a condensed 532 nm light beam from the bottom over time, where the laser is moved from position A to B to C. (b) Sum-up of the crack’s deviation due to the laser’s moving.


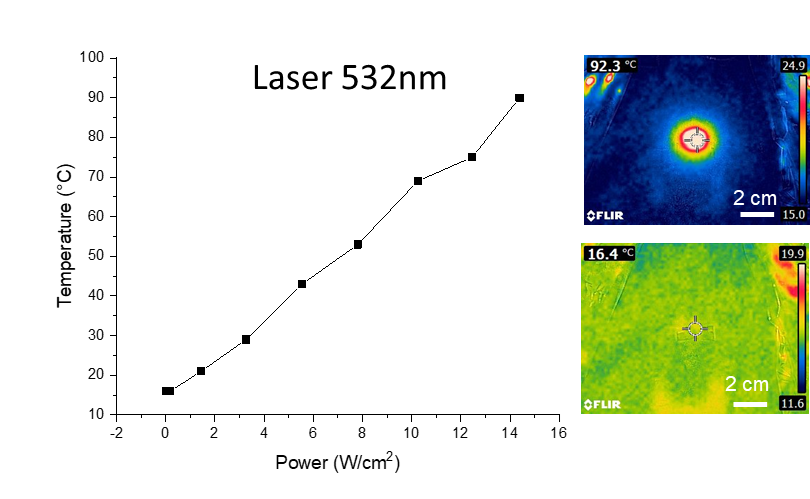


Supplementary Figure 9: Characterization of the photo-thermal properties of a plasmonic substrate. Temperature evolution of a plasmonic substrate (dewetted gold particles) as function of the 532 nm laser power density measured with an infrared camera. Source data are provided as a Source Data file.


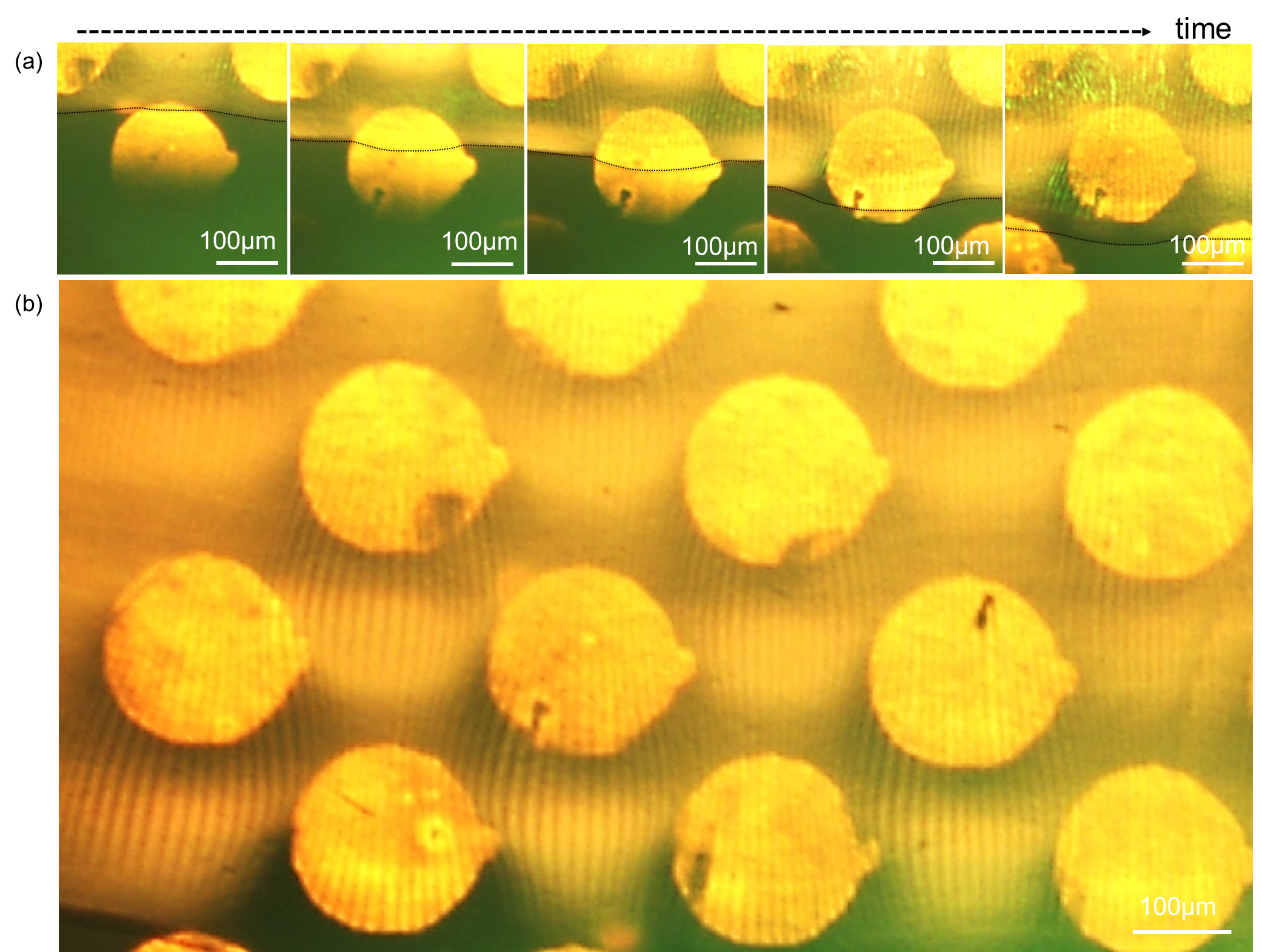


Supplementary Figure 10: Evolution of the drying front line. (a) Snapshots of the evolution of the drying front line when it encounters a plasmonic spot (yellow circle), illuminated by a 532 nm laser. (b) Optical microscopy image of the final light-deviated cracked pattern.


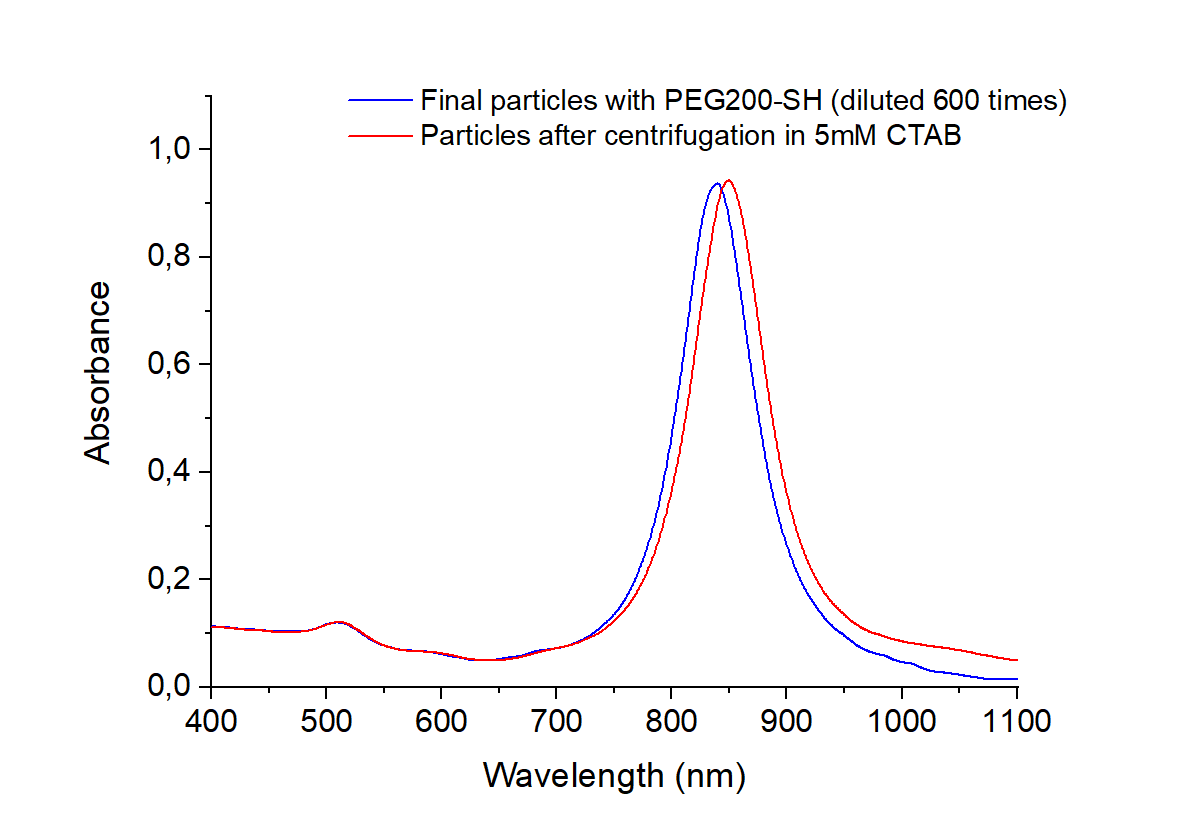


Supplementary Figure 11: Characterization of AuBP. Spectra of AuBP@840 nm PEG 2000 before and after centrifugation in a CTAB solution. Source data are provided as a Source Data file.


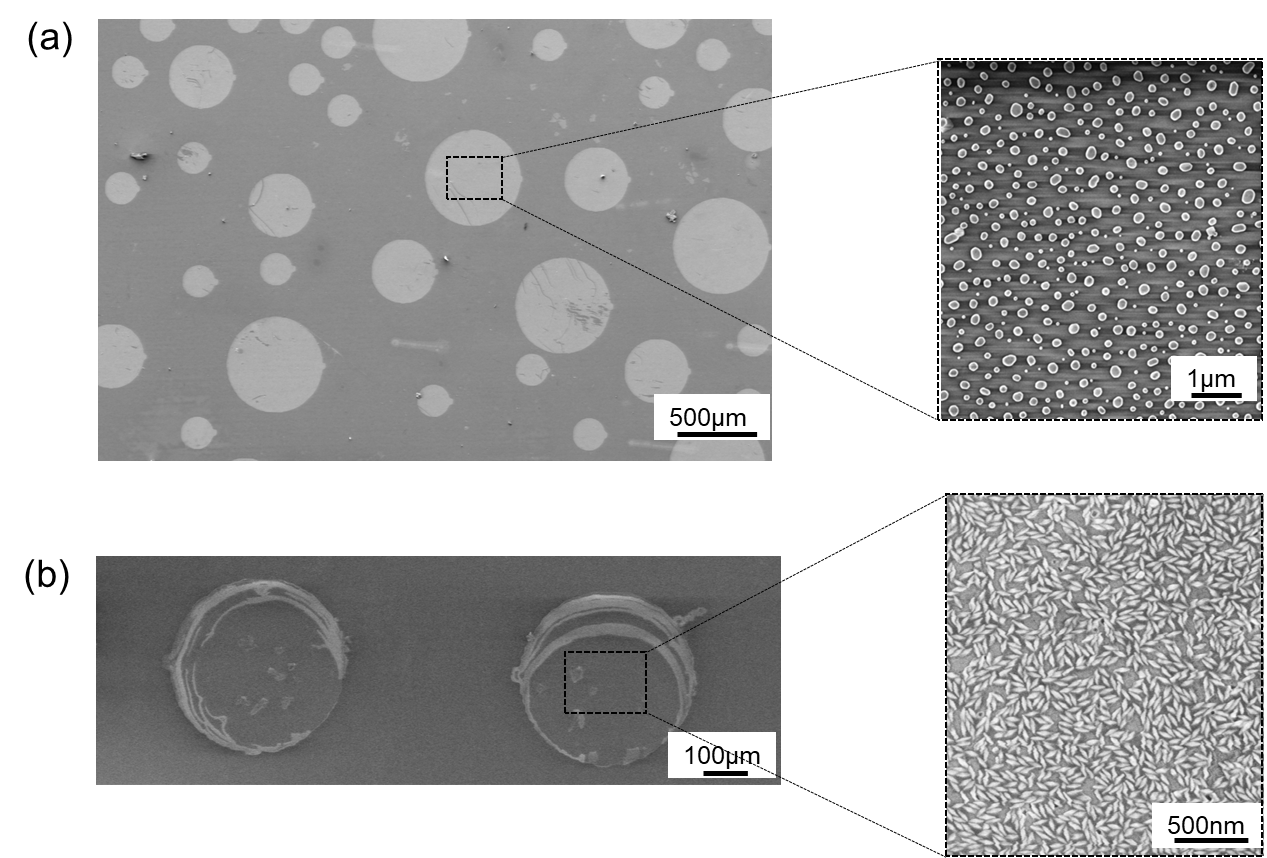


Supplementary Figure 12: SEM characaterization of plasmonic substrates. SEM images of patterns obtained (a) by dewetting gold particles, and (b) by deposition of gold bipyramids.


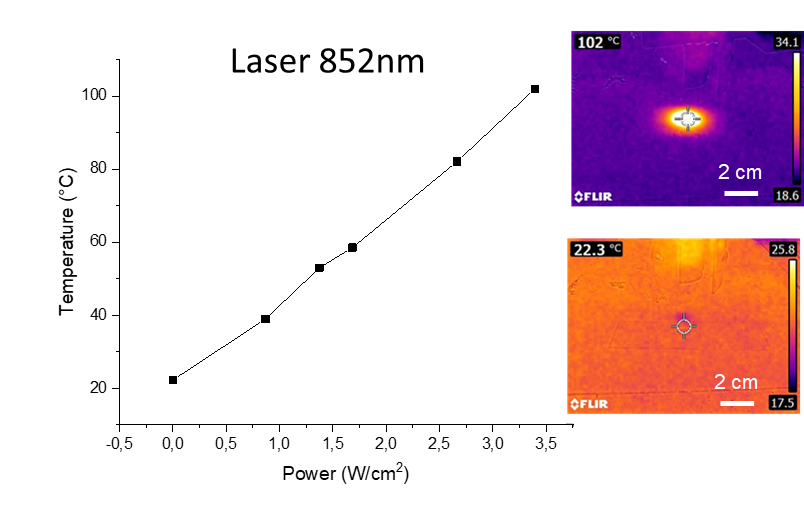


Supplementary Figure 13: Characterization of the photo-thermal properties of a plasmonic colloidal solution.Temperature evolution of a PS colloidal solution containg the AuBPs as function of the 852 nm laser power density measured with an infrared camera. Source data are provided as a Source Data file.


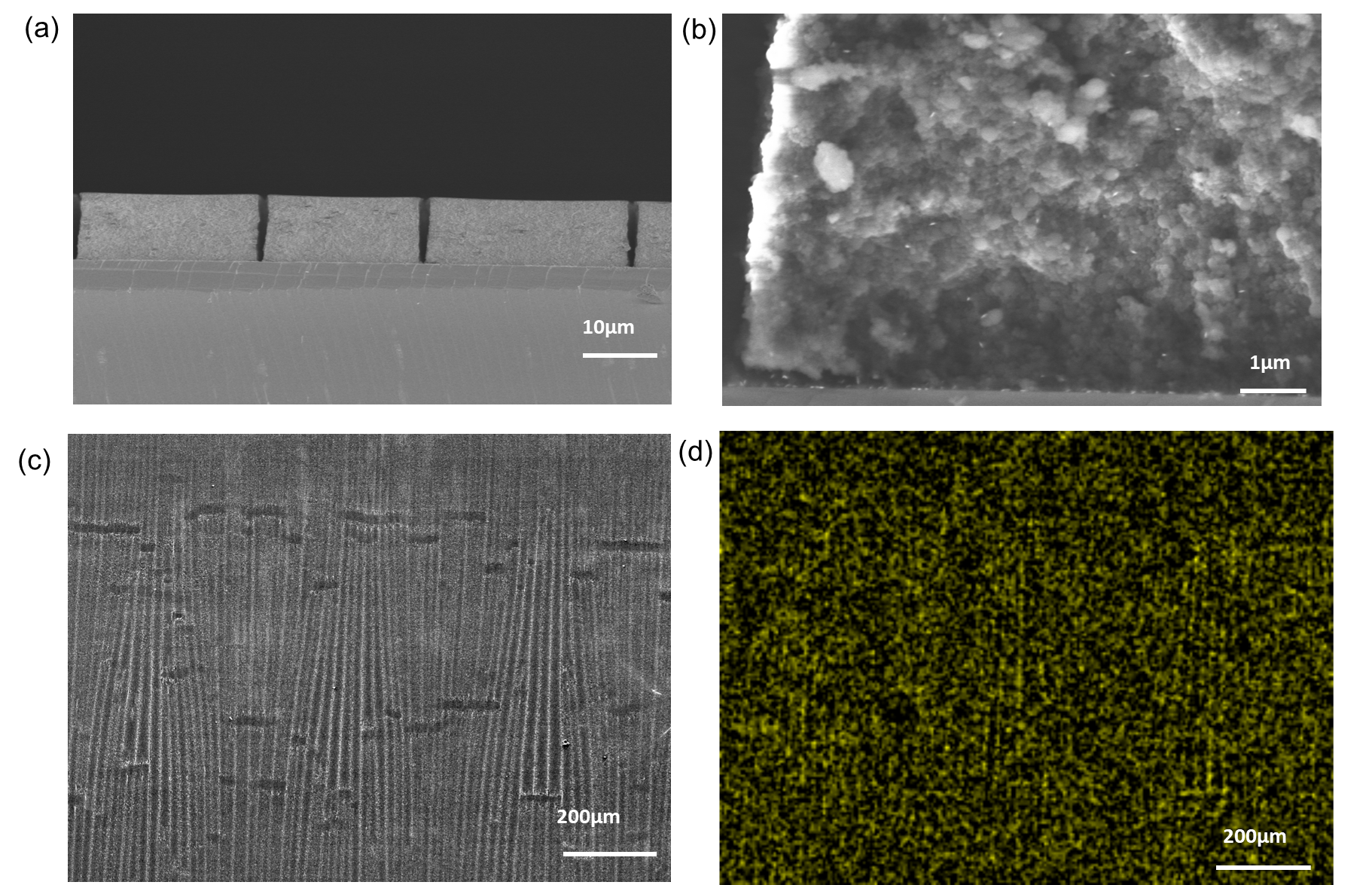


Supplementary Figure 14: Characterization of the repartition of AuBP in a colloidal film. (a) Cross-section SEM image of deviated cracked layer, with a (b) zoomed-in view of the distribution of spherical polystyrene nanoparticles and AuBPs (brighter spots). (c) SEM top-view image of a deviated crack film, with corresponding (d) Energy Dispersive X-Ray Spectroscopy analysis, showing the distribution of gold particles.


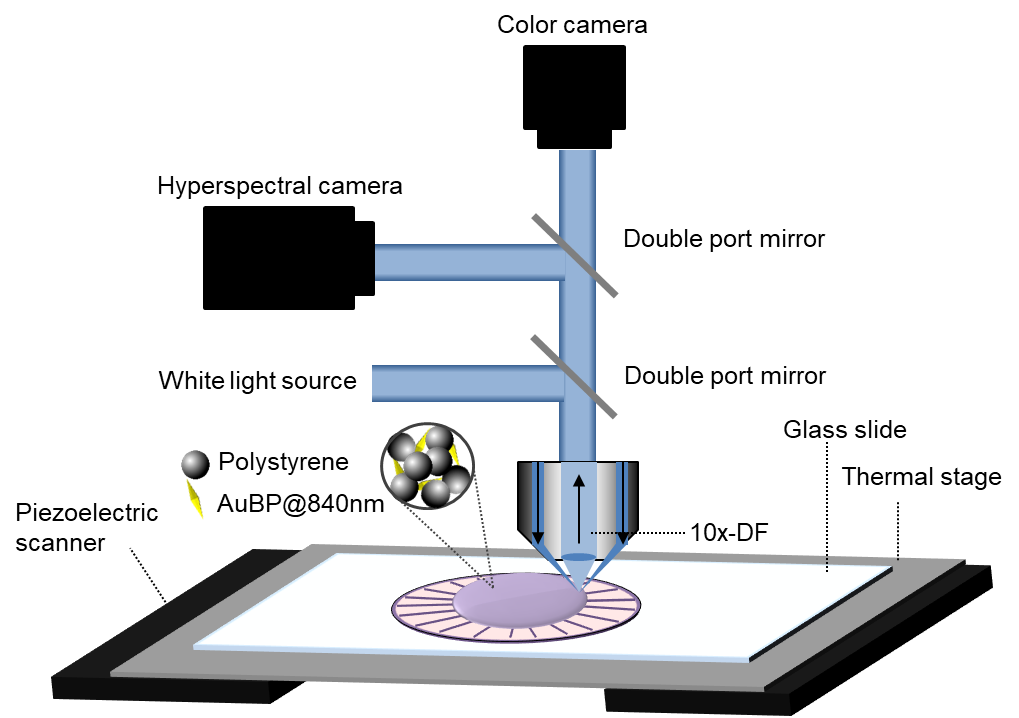


Supplementary Figure 15: Scheme of the experimental set-up of hyperspectral microscope to analyze the evolution of the plasmonic peak during the drying of a colloidal droplet. The measurement was taken in dark field mode with a x10 objective in reflection.


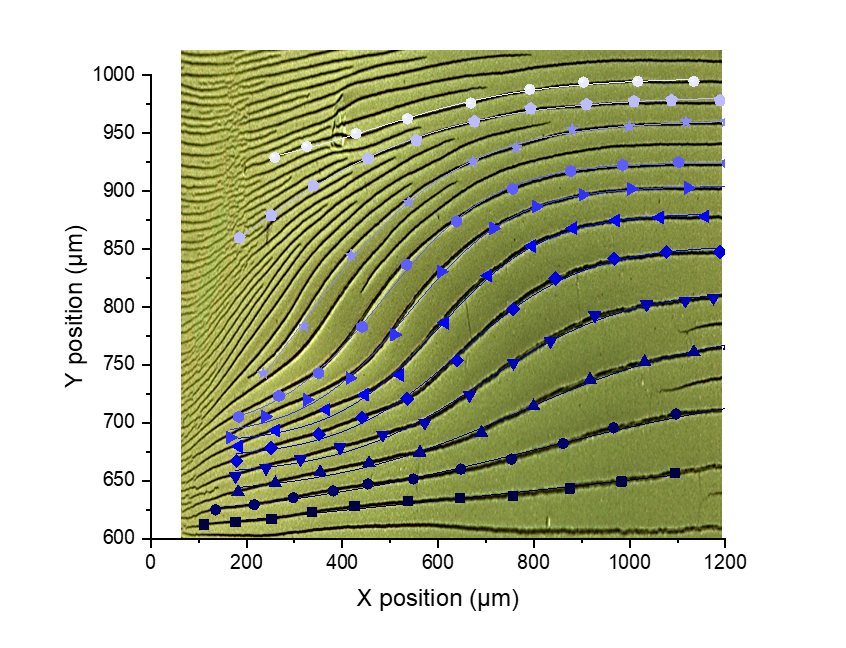


Supplementary Figure 16: Image analysis of the crack deviation by Boltzmann-like equation.

$A_{1}$and $A_{2}$ represent the y position of the crack at the beginning and the end of the deviation respectively, and $x_{0}$ the x position of the centre where the middle of the slop is reached.

$y=\frac{A_{1}-A_{2}}{1+e^{\frac{(x-x_{0})}{dx}}}+ A_{2}$ (1)

Thus, two parameters are obtained to compare the different microscope images, namely the angle of deviation, meaning the slop ($y^{'}=\frac{A_{1}-A_{2}}{4dx}$ ) and the difference of deviation ($delta A= A_{2}-A_{1}$).


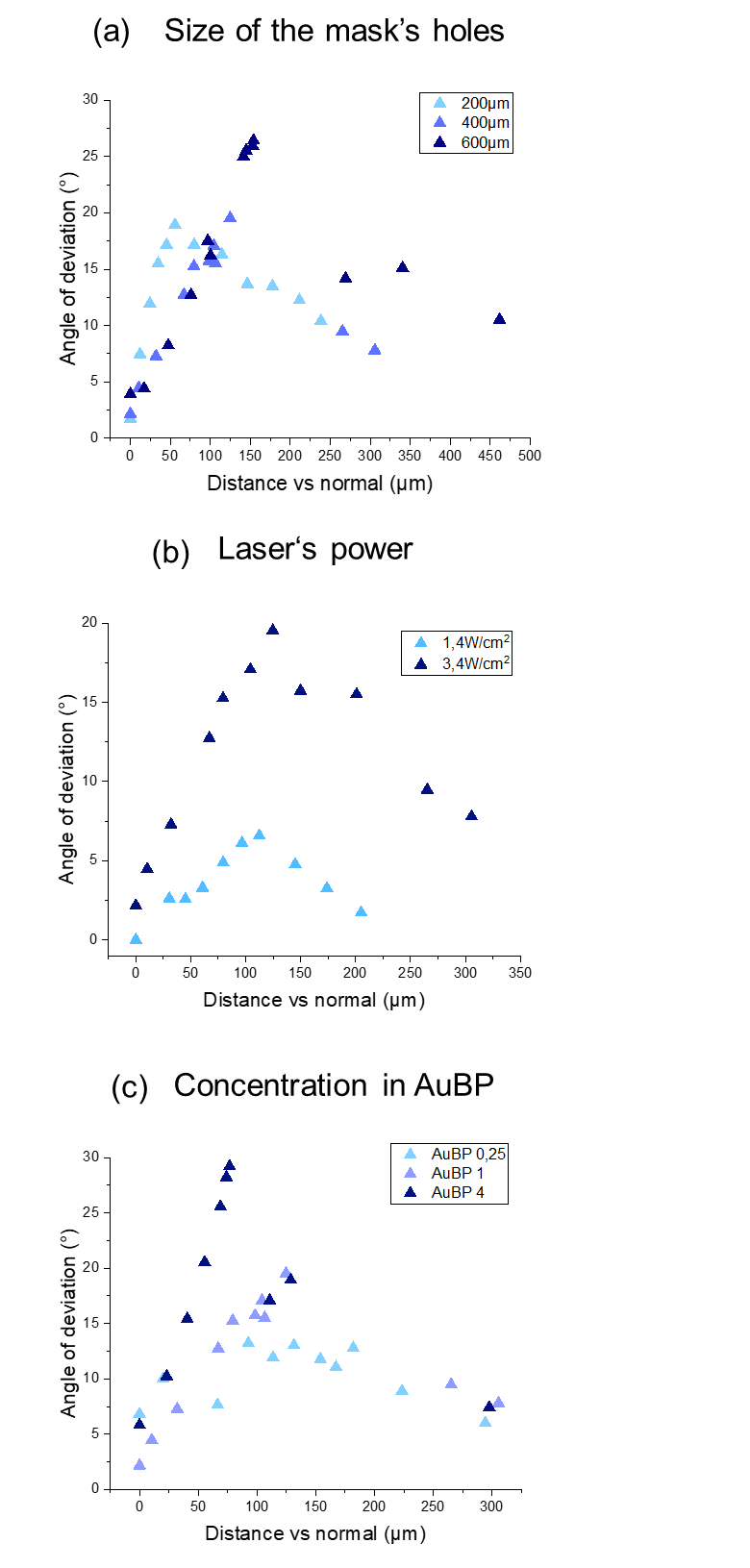


Supplementary Figure 17: Analysis of the angle of deviation as function of the distance versus the normal, depending of the (a) the size of the hole of the mask, (b) the laser’s power and (c) the concentration in bipyramids. Source data are provided as a Source Data file for each figures.


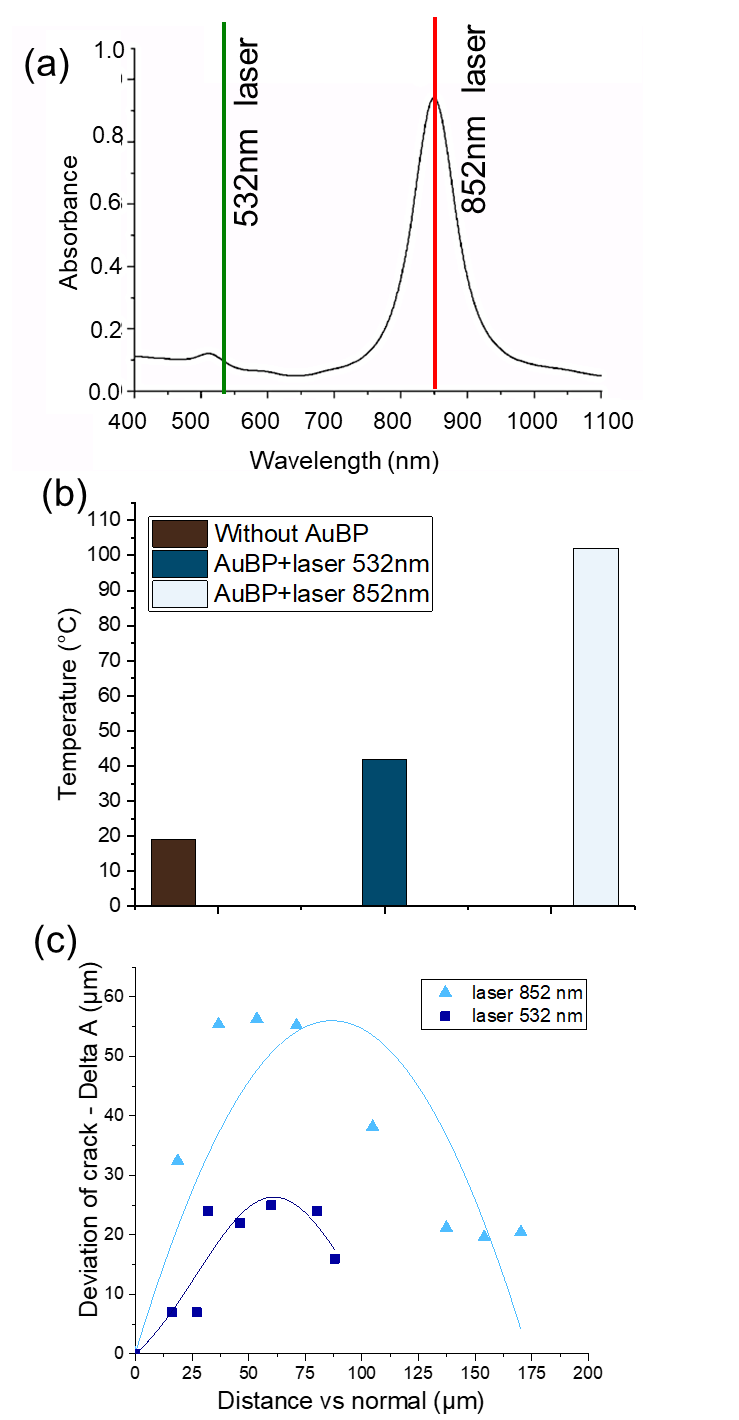


Supplementary Figure 18: Sensitivity of AuBP to different lasers. (a) Absorption spectrum of colloidal Au bipyramids, which exhibit two absorption peaks: a prominent longitudinal absorption peak at around 850 nm and a weaker transverse absorption peak at approximately 515 nm. (b) Temperature evolution of the AuBPs/PS solution irradiadiated with a 532 nm or 852 nm laser (power 3.4 W/cm²). The temperature of the PS colloidal solution without AuBPs and irradiated with a laser at 852 nm (power 3.4 W/cm²) is reported as reference. (c) Analysis of the deviation of cracks as function of the distance versus the normal, depending of the laser wavelength. Source data are provided as a Source Data file for Figure (c).


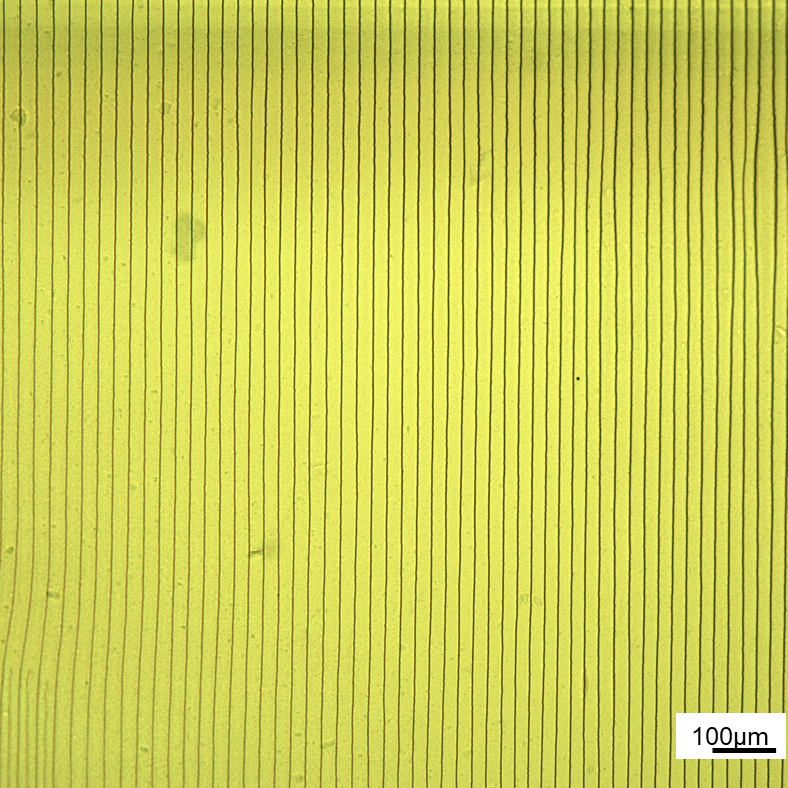


Supplementary Figure 19: Optical micrograph of cracks obtained by colloidal solution without AuBPs (852 nm irradiation).


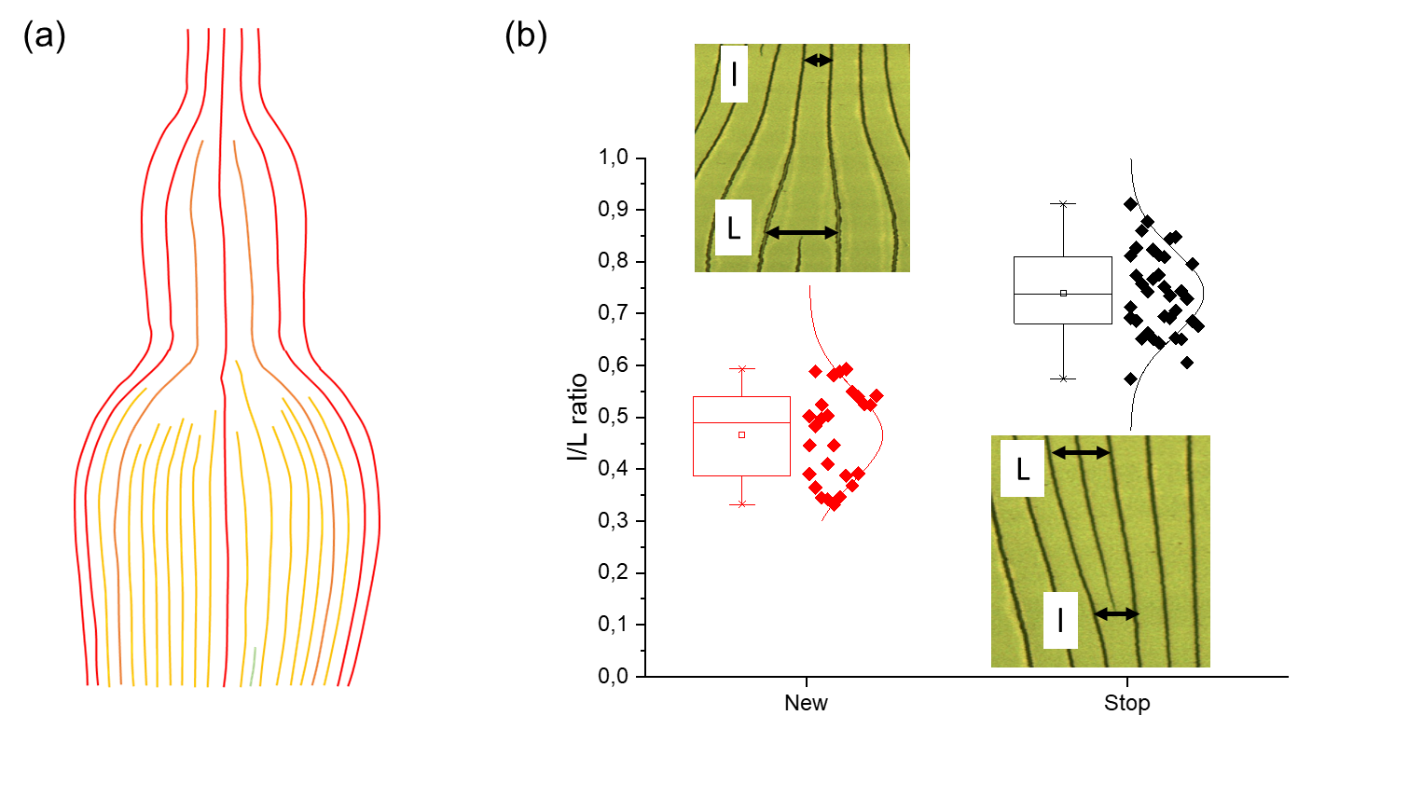


Supplementary Figure 20: Formation of new crack or stop of an existing one as function of the divergence or convergence ratios as illustrated in the insets. The divergence ratio is defined as the l/L ratio where l is distance between two adjacent cracks before deviation and L the distance between the two cracks when a new crack appears. The convergence ratio is defined as the l/L ratio where l the distance between the two adjacent cracks when a middle crack arrests and L is the distance between same cracks before deviation.


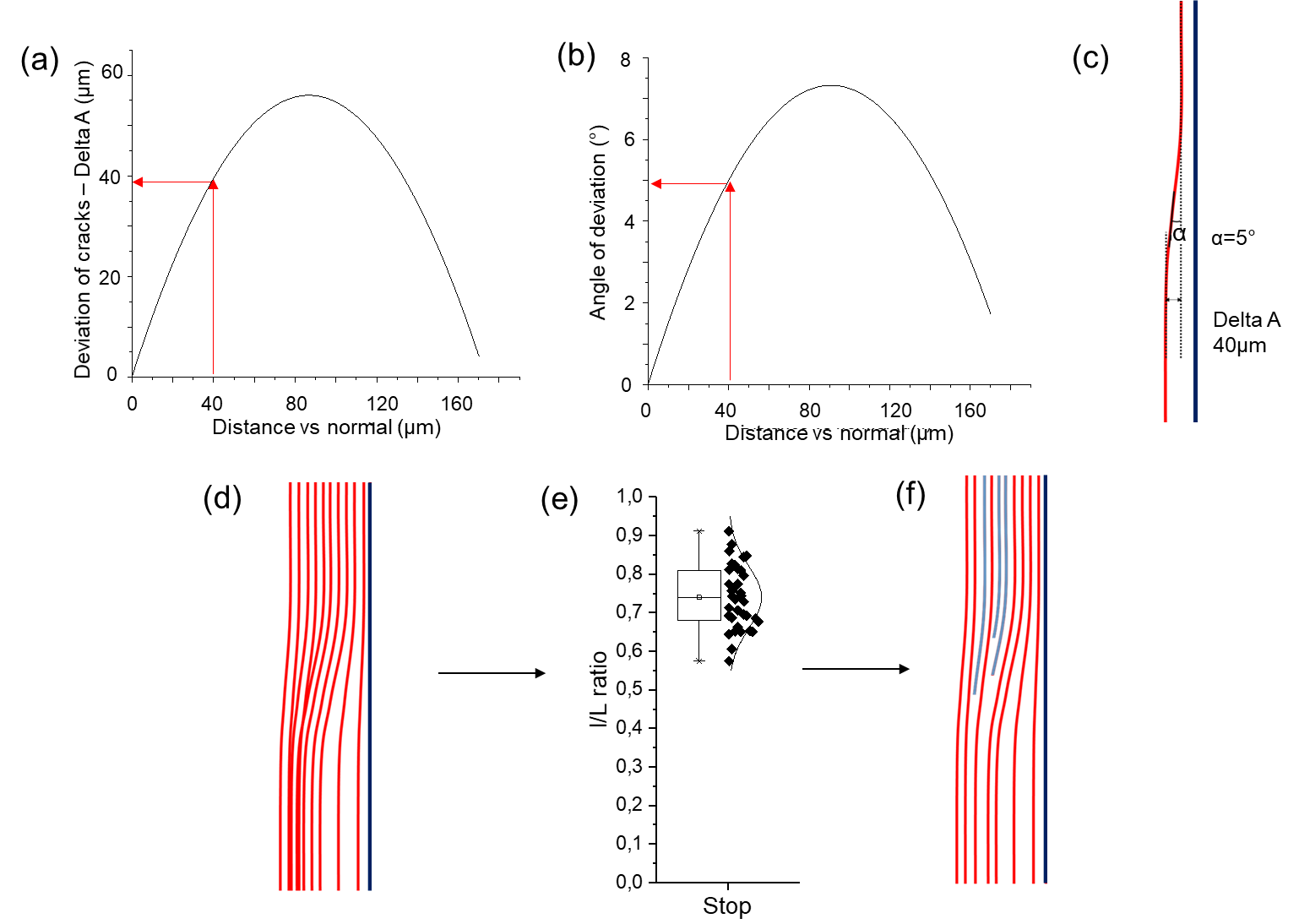


Supplementary Figure 21: Programmed crack pattern. The experimentally measured deviation of crack (Delta A – (a)) and angle of deviation (α – (b)) were described by a Gaussian function. (c) For a give distance from the center, the deviation and angle of the first crack can be drawn. (d) Subsequently, this process is repeated several times for periodic cracks at increasing distances. In some cases, cracks converge. However, in a reality, such phenomenon cannot occur, as certain cracks will stop when they converge. (e) Based on our experimental findings, we determined this convergence ratio ratio to be 0.74. (f) Consequently, the last step consists of arreting cracks that are too close to each other.


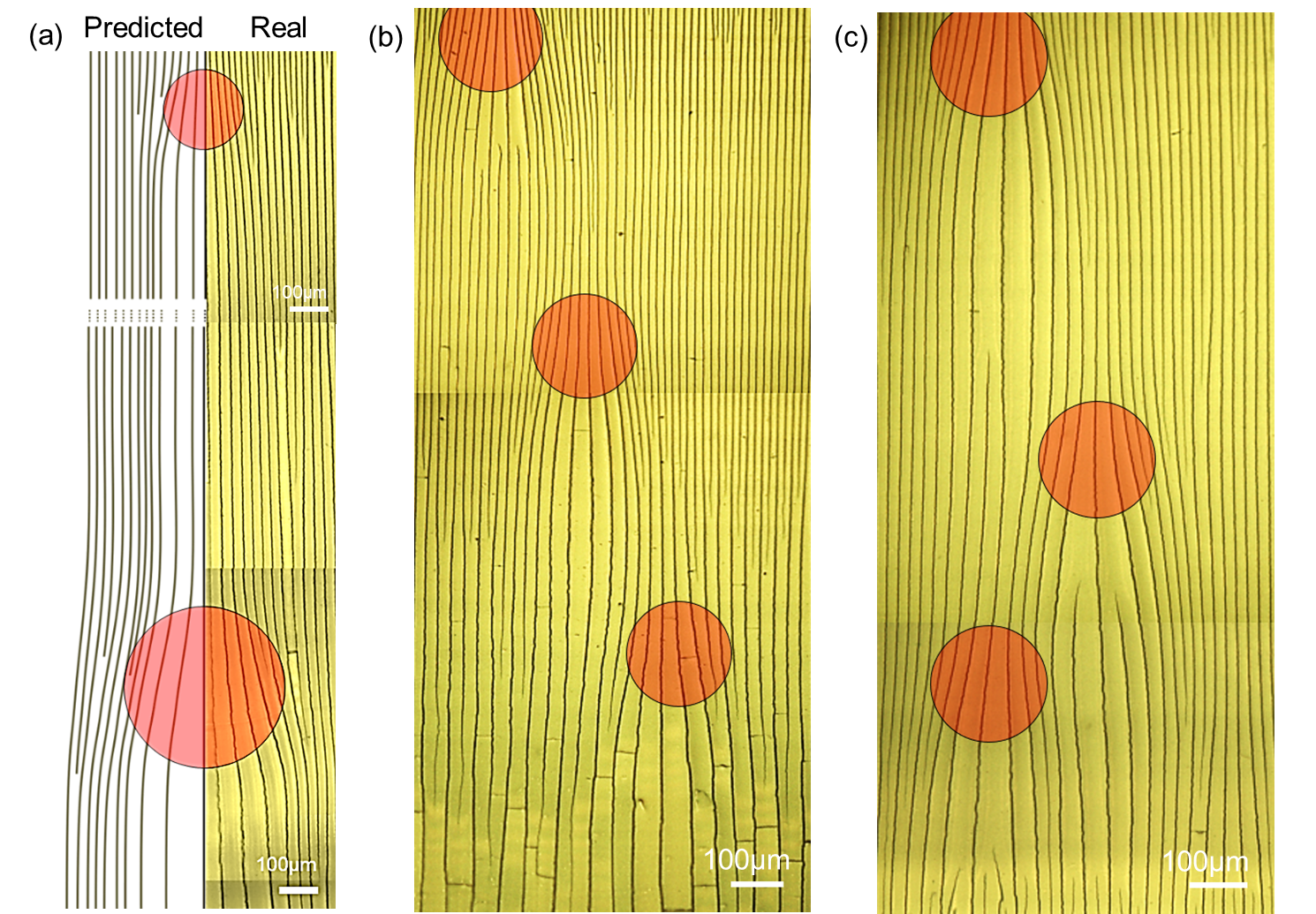


Supplementary Figure 22: Examples of curved patterns with distinct morphologies (a) Expected and real experimental pattern obtained for two circular spots of 200 µm diameter and 400 µm diameter placed one above the other. (b) and (c) optical microscopy image of cracked patterns obtained with a plasmonic colloidal solution. Red spots represent where the laser hits the plasmonic solution.


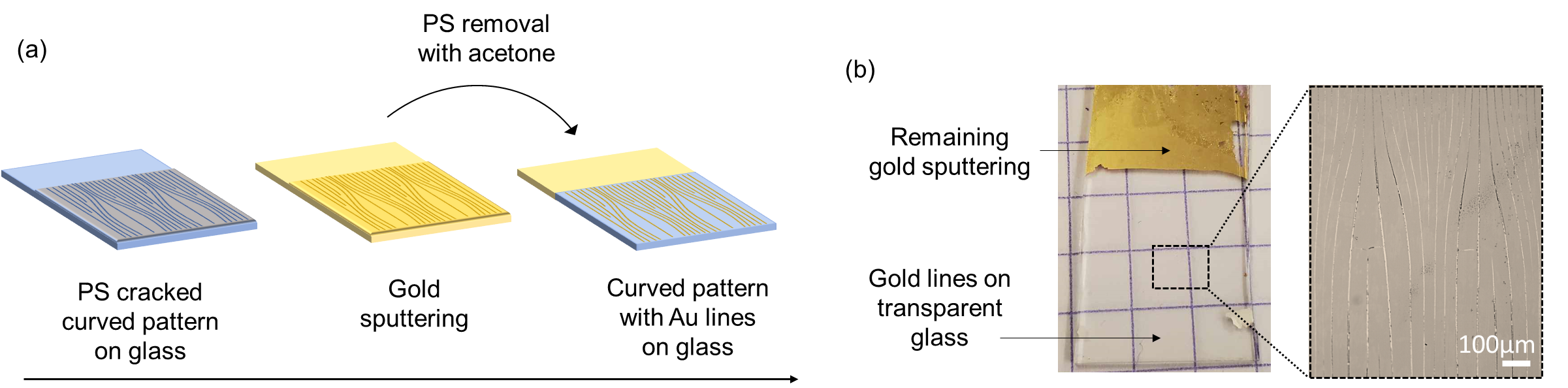


Supplementary Figure 23: Fabrication of curved gold lines. (a) Scheme of the lift-off process in order to obtain Au lines starting from PS cracked pattern. (b) Photograph of a sample with the gold curved lines on a glass slide and its optical microscopy image.

Supplementary Figure 24: Generalization of the self-ordering process to other colloidal systems. Optical micrographs of oriented periodic cracks in YAG:Ce, SiO_2_ and PMMA colloidals films. The water based colloidal solution were obtained from protocols from the literature ^1,2^.

**Supplementary References**

1. Odziomek, M., Chaput, F., Lerouge, F., Sitarz, M. & Parola, S. Highly luminescent YAG:Ce ultra-small nanocrystals, from stable dispersions to thin films. *J. Mater. Chem. C* **5**, 12561–12570 (2017).

2. De Marco, M. L. *et al.* High-Entropy-Alloy Nanocrystal Based Macro- and Mesoporous Materials. *ACS Nano* **16**, 15837–15849 (2022).
