# Supplementary material for: Programming crack patterns with light in colloidal plasmonic films
Source: Nat Commun. 2024 Feb 7;15:1156. doi: 10.1038/s41467-024-45365-1 (PMC10850101; doi:10.1038/s41467-024-45365-1)
Supplement: Supplementary file 3 — Description of Additional Supplementary Files [file 41467_2024_45365_MOESM3_ESM.docx]

**Description of Additional Supplementary File**

**File Name: Supplementary Movie 1**
**Description**: Drying of a droplet of an aqueous solution of polystyrene colloids on a plasmonic substrate and illuminated by a 532 nm-laser (532 nm) at a power of 0.19 W/cm^2^.

**File Name: Supplementary Movie 2
Description**: Drying of a droplet of an aqueous solution of polystyrene colloids on a plasmonic substrate and illuminated by a 532 nm-laser (532 nm) at a power of 0.67 W/cm^2^.

**File Name: Supplementary Movie 3**
**Description**: Drying of a droplet of an aqueous solution of polystyrene colloids on a substrate without plasmonic particles. The cracks are not deviated when illuminated with the laser.

**File Name: Supplementary Movie 4
Description**: Manipulation of crack propagation when illuminated by a laser beam at different positions.

**File Name: Supplementary Movie 5
Description**: Drying of an aqueous solution of polystyrene colloids by dip-coating on a patterned plasmonic substrate and illuminated by a 532nm-laser.
